# Non-invasive methods for estimating mPAP in COPD using cardiovascular magnetic resonance imaging

**DOI:** 10.1007/s00330-017-5143-y

**Published:** 2017-11-16

**Authors:** C. S. Johns, S. Rajaram, D. A. Capener, C. Oram, C. Elliot, R. Condliffe, D. G. Kiely, J. M. Wild, A. J. Swift

**Affiliations:** 1 Academic Unit of Radiology, C floor, Royal Hallamshire Hospital, Glossop Rd, Sheffield, S10 2JF UK; 2 Sheffield Pulmonary Vascular Disease Institute, Sheffield, UK; 3 Insigneo, Institute of In-Silico Medicine, Sheffield, UK

**Keywords:** Pulmonary Hypertension, Cardiac, Magnetic Resonance Imaging, Chronic Obstructive Pulmonary Disease, Cor Pulmonale

## Abstract

**Purpose:**

Pulmonary hypertension (PH) is associated with a poor outcome in chronic obstructive pulmonary disease (COPD) and is diagnosed invasively. We aimed to assess the diagnostic accuracy and prognostic value of non-invasive cardiovascular magnetic resonance (CMR) models.

**Methods:**

Patients with COPD and suspected PH, who underwent CMR and right heart catheter (RHC) were identified. Three candidate models were assessed: 1, CMR-RV model, based on right ventricular (RV) mass and interventricular septal angle; 2, CMR PA/RV includes RV mass, septal angle and pulmonary artery (PA) measurements; 3, the Alpha index, based on RV ejection fraction and PA size.

**Results:**

Of 102 COPD patients, 87 had PH. The CMR-PA/RV model had the strongest diagnostic accuracy (sensitivity 92%, specificity 80%, positive predictive value 96% and negative predictive value 63%, AUC 0.93, *p*<0.0001). Splitting RHC-mPAP, CMR-RV and CMR-PA/RV models by 35mmHg gave a significant difference in survival, with log-rank chi-squared 5.03, 5.47 and 7.10. RV mass and PA relative area change were the independent predictors of mortality at multivariate Cox regression (*p*=0.002 and 0.030).

**Conclusion:**

CMR provides diagnostic and prognostic information in PH-COPD. The CMR-PA/RV model is useful for diagnosis, the RV mass index and PA relative area change are useful to assess prognosis.

***Key Points*:**

• *Pulmonary hypertension is a marker of poor outcome in COPD*.

• *MRI can predict invasively measured mean pulmonary artery pressure*.

• *Cardiac MRI allows for estimation of survival in COPD*.

• *Cardiac MRI may be useful for follow up or future trials*.

• *MRI is potentially useful to assess pulmonary hypertension in patients with COPD*.

## Background

Pulmonary hypertension is a predictor of death and hospitalisation [1–5] in patients with chronic obstructive pulmonary disease (COPD). Patients with pulmonary hypertension in COPD, as defined by a mean pulmonary artery pressure (mPAP) of ≥25 mmHg have a 5-year survival rate of 36% [6]. A number of studies in patients with severe COPD have shown that mild pulmonary hypertension is common: a recent study in patients with severe COPD referred for lung volume reduction surgery demonstrating pulmonary hypertension at right heart catheter in 50% [7, 8]. More recently, with the advent of therapies for other forms of pulmonary hypertension there has been increasing interest in the subset of lung disease patients with severe (previously called “out of proportion”) pulmonary hypertension, defined as an mPAP ≥35 mmHg or mPAP ≥35 mmHg with cardiac index ≤2.0 [9], where a cardiovascular limitation to exercise, rather than respiratory limitation exists [10]. This raises the possibility that pulmonary vascular therapies may improve symptoms and outcome in this patient group [11].

The gold standard for diagnosis of pulmonary hypertension is right heart catheter (RHC), however, this is an invasive test [12]. As such, patients are screened for pulmonary hypertension with echocardiography, but unfortunately, this is less accurate in COPD where pulmonary artery pressure, when measured, is often overestimated [13, 14]. A non-invasive method for estimating mPAP in COPD patients would, therefore, be useful to help diagnose PH, in prognostication and for possible assessment of treatment response or follow-up in clinical trials.

Several predictive cardiac magnetic resonance (CMR) imaging models have been proposed for estimation of pulmonary artery pressure [15–17]. The predictive value of these MR derived imaging models in a population of patients with suspected pulmonary hypertension in COPD remains unknown. The aim of this study was to assess the diagnostic accuracy and the prognostic value of these published models of non-invasive mPAP prediction using cardiovascular MRI.

## Materials and Methods

All consecutive patients who underwent MRI at a pulmonary hypertension referral centre [18] from April 2012 to October 2015 with suspected pulmonary hypertension were assessed for inclusion. Inclusion criteria were a formal diagnosis of COPD (according to standard criteria), as per the ASPIRE (Assessing the Spectrum of Pulmonary hypertension Identified at a REferral centre) registry [18]. Patients were assessed for either obstructive spirometry (defined as FEV1/FVC ratio of ≤0.70) or qualitative CT evidence of emphysema, as per standard radiological practice [19]. Any patients without RHC and MRI within 90 days were excluded. Ethical approval was granted from a local ethics committee for this retrospective study, written consent was waived (ref c06/Q2308/8).

### Image Acquisition

Cardiac MRI was performed in a pulmonary hypertension tertiary referral centre [18], on a GE HDx 1.5-T whole body scanner (GE Healthcare, Milwaukee, WI, USA), using an 8-channel cardiac coil, with the patient supine. Four-chamber (4Ch) and short axis (SA) cine images were acquired using a retrospectively cardiac gated multi-slice steady-state free precession (SSFP) sequence. A stack of axial images in the SA plane with slice thickness of 8 mm with a 2 mm inter-slice gap or 10 mm with no inter-slice gap were acquired, covering both ventricles from base to apex. The SSFP sequence parameters were: TR 2.8 ms, TE 1.0 ms, flip angle 50°, field of view 48x43.2, 256x256 matrix, 125 kHz bandwidth, and slice thickness 8 to 10 mm. This cardiac MRI scan protocol takes approximately 40 min to perform.

### Image Analysis and Metrics

**Fig. 1:**
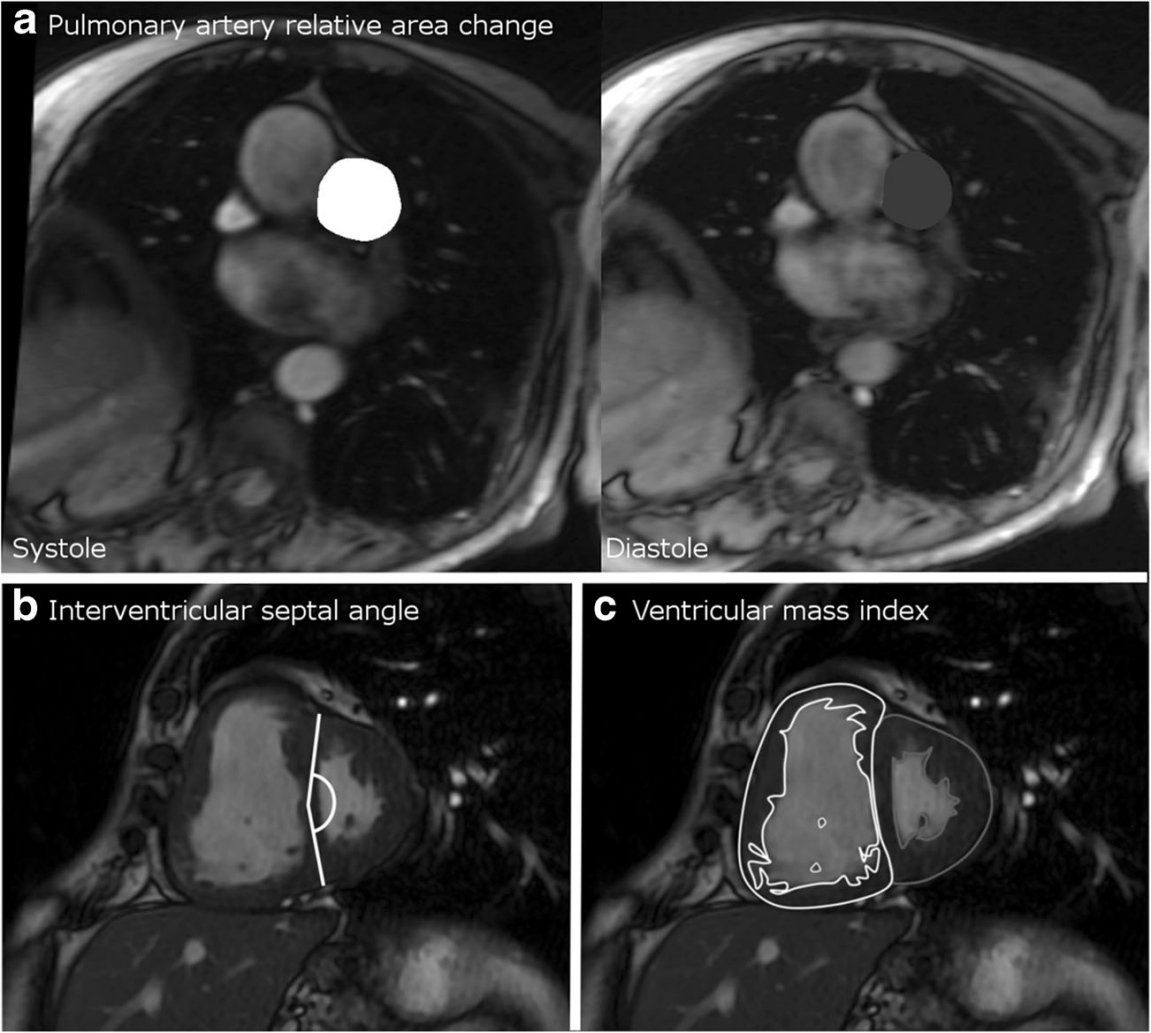
Diagram showing the methodology of calculation of the cardiac MRI metrics. Pulmonary artery relative area change (**a**) taken from cine images taken at the level of the pulmonary trunk, perpendicular to the main pulmonary artery; septal angle (**b**) taken as the angle made between the insertion points of the RV to the mid septum on the end-systolic image from the short axis stack; and (**c**) ventricular mass index taken by segmentation of the muscle mass of the left and right ventricle on the stack of images taken in the short axis plane

MR images were manually analysed by DC (a cardiac MRI radiographer of 9 years cardiac MRI experience) on a GE Advantage Workstation 4.4 and GE Advantage Workstation ReportCard software, with the observer blinded to all clinical information and other investigations. Left and right ventricular end-diastolic volume, end-systolic volume, right and left ventricular stroke volume and mass were calculated (all indexed to body surface area), right and left ventricular ejection fraction, ventricular mass index (RV mass divided by LV mass) [20] and interventricular septal angle were measured as previously described [15]. Right and left ventricular ejection fractions were calculated as (end diastolic volume minus end-systolic volume) divided by end diastolic volume. Maximal and minimal PA areas were manually traced, and relative area change was defined by the following equation: RAC= (maximum area-minimum area/minimum area) [21]. Reproducibility metrics for these cardiac MRI metrics for DC and AJS have been previously published [22]. Please see Fig. 1 for a diagram of the key imaging metrics that were measured.

### CMR image based models

Previously published parametric models, developed for diagnostic and prognostic assessment in PH, were derived from cardiac MRI metrics:

1. The CMR-RV model is based on ventricular mass index and interventricular septal angle: mPAP = –4.6 + (interventricular septal angle x 0.23) + (ventricular mass index x 16.3), see Fig. 1 (A and B). This model was developed in a cohort of 64 treatment naïve patients with suspected pulmonary hypertension in a tertiary referral centre. In a derivation cohort of 64 patients with suspected pulmonary hypertension, this showed good correlation with right heart catheter measured mPAP and strong diagnostic accuracy. The published threshold of ≥32 mmHg had 87% sensitivity and 90% specificity for the presence of all causes of pulmonary hypertension [15].
2. The PA/RV model is similar to the CMR-RV model (above), with the addition of basic function metrics of the pulmonary artery: CMR-PA/RV = -21.806 + (inter-ventricular septal angle x 0.31) + (ventricular mass index x 11.5) + (diastolic pulmonary artery area x 0.01) – (PA relative area change x 0.22), see Fig. 1 (A, B, C and D). This model was derived in 247 patients with suspected pulmonary hypertension patients and was predictive of pulmonary hypertension in a separate prospective cohort of 115 patients, with an area under the receiver operator curve of 0.92 [16].
3. The alpha index comprises both functional and structural information, utilising right ventricular function along with pulmonary artery size: Alpha index = minimum PA area/RV ejection fraction. This model was assessed in a cohort of 185 patients, with an area under ROC curve of 0.95 [17].

The diagnostic cut-off for pulmonary hypertension for CMR-RV was ≥32 and alpha index ≥7.2, as published in the literature [15–17]. CMR-PA/RV does not have a published threshold, so we used a threshold of ≥25, as this mirrors the threshold of invasively measured mPAP at right heart catheterisation.

### Right Heart Catheterisation

Right heart catheterisation was performed using a balloon tipped 7.5 Fr thermodilution catheter (Becton-Dickinson, Franklin Lakes, NJ, USA). Pulmonary hypertension was defined as a resting mPAP ≥25 mmHg and severe pulmonary hypertension as a resting mPAP ≥35 mmHg. Cardiac output was calculated using thermodilution.

### Statistics

Pearson’s correlation between CMR models and RHC-mPAP were calculated. The relative accuracy of the models was assessed using Bland-Altman plots. Diagnostic accuracy was calculated from the 2x2 contingency table to calculate sensitivity, specificity, negative predictive value and positive predictive value. Receiver operating characteristic (ROC) curves were constructed and the area under the ROC curve (AUC) recorded.

The interval from CMR until all cause death or census was regarded as the follow-up period. The census was performed on 25/02/2016. Log-log plots were visually inspected to ensure linearity with outcome data. Survival analysis was performed using univariate and multivariate Cox proportional hazards regression. In order to allow for comparison between each metric, this was performed with each variable standardised as the z score for the population studied. Multivariate analysis was performed in a forward direction, for all variables with a statistically significant association with mortality on univariate analysis.

Kaplan-Meier plots were generated for each model and RHC measured mPAP, dichotomised by a value of 35, and Chi square values were calculated using the Log rank test, as this is the threshold value for severe pulmonary hypertension [9]. Statistical analysis was made using SPSS 22 (IBM, Chicago, IL, USA) and graphs were created using GraphPad Prism 7 (GraphPad Software, San Diego, CA, USA). A *p*-value of <0.05 was considered statistically significant.

## Results

**Fig. 2:**
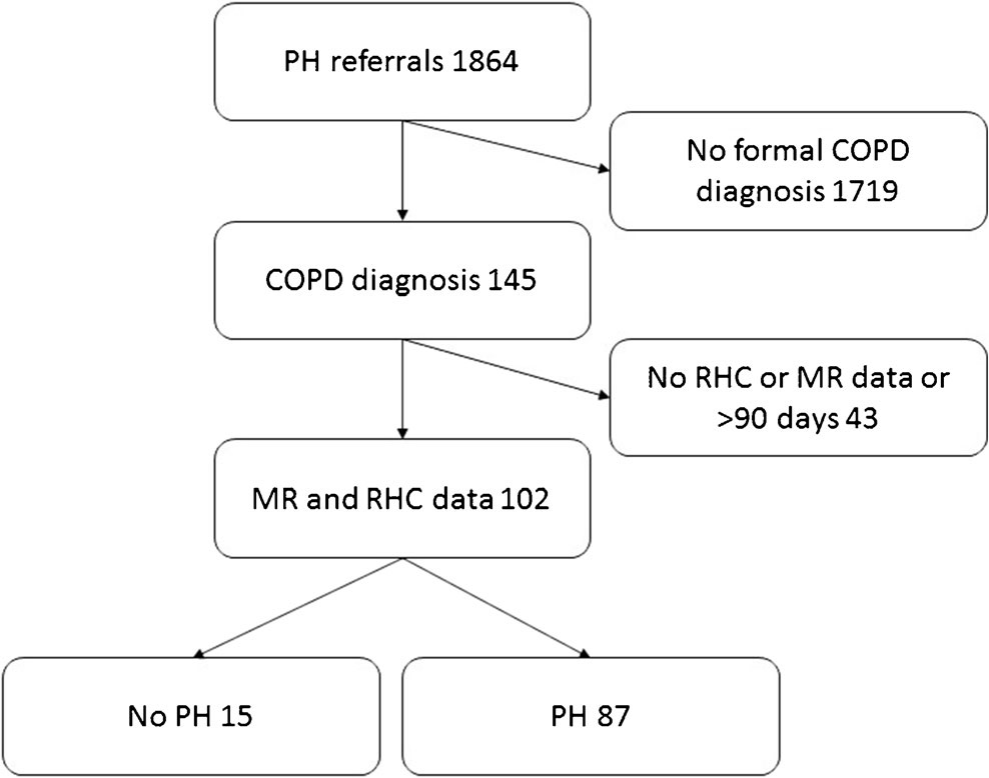
Patient selection

A total of 1864 patients were referred to the pulmonary hypertension centre during the period studied and underwent right heart catheterisation: of these 145 had a documented diagnosis of COPD from a respiratory specialist. One hundred and two had MRI and RHC within 90 days so were included in the study. There were 87 patients with pulmonary hypertension (69 of these had severe pulmonary hypertension) and 15 patients without pulmonary hypertension (please see Fig. 1 for the patient diagnostic pathway). Figure 2 shows the flow of patients within the diagnostic pathway. There were 24 with GOLD severity 1, 40 with GOLD severity 2, 18 with GOLD severity 3 and seven with GOLD severity 4; 13 patients did not have spirometry results available for analysis. Of the 102 cases of COPD included in the study, 75 had spirometric evidence of airflow limitation (FEV1/FVC ratio <0.7), and the remaining 17 had evidence of emphysema on CT. Table 1 shows the patient demographics and clinical characteristics for the whole group and split into no PH (mPAP <25 mmHg), PH (mPAP 25 to ≤35 mmHg) and severe PH (mPAP ≥35 mmHg).

**Table 1:** Patient demographics, mean with standard deviation with ANOVA p-value between no PH, mild PH and severe PH

	All patients	No PH	PH	Severe PH	*p*-value (ANOVA)
Number	102	15	18	69	
Clinical Demographics					
Age (years)	67 (12)	59 (17)	67 (12)	69 (9)	0.012
Sex (F/M)					
WHO functional class (II,III,IV)					
ISWT (m)	174 (169)	432 (264)	141 (101)	121 (87)	<0.001
Baseline catheter data					
mPAP (mmHg)	40 (12)	21 (3)	31 (3)	47 (8)	<0.001
mRAP (mmHg)	11 (6)	6 (2)	8 (3)	11 (7)	0.056
PAWP (mmHg)	12 (4)	10 (3)	12 (3)	13 (4)	0.013
CI (Litre/m^2^)	2.8 (0.9)	3.1 (0.7)	3.2 (0.9)	2.6 (0.9)	0.005
PVRI (Dyne.s)	3.7 (0.9)	3.1 (0.7)	3.2 (0.9)	2.6 (0.9)	<0.001
SvO2 (%)	65 (9)	72 (8)	69 (5)	62 (9)	<0.001
Days RHC to MR	5 (15)	5 (19)	5 (13)	5 (14)	0.989
Spirometry	n=89	n=9	n=17	n=63	
FEV1 (% Pred)	66 (24)	76 (24)	58 (15)	66 (25)	0.165
FVC (% Pred)	85 (22)	92 (20)	77 (15)	87 (24)	0.155
FEV1/FVC	0.55 (0.15)	0.63 (0.20)	0.51 (0.10)	0.55 (0.15)	0.179
TLCO (% Pred)	30 (22)	80 (31)	33 (15)	21 (9)	<0.001
Cardiac MRI					
RV EDV index	89 (35)	72 (30)	70 (32)	97 (34)	0.002
RV ESV index	56 (31)	36 (18)	37 (26)	66 (30)	<0.001
RVEF	40 (14)	51 (8)	51 (11)	35 (12)	<0.001
RV mass index					
LV EDV index	55 (18)	62 (10)	63 (18)	52 (19)	0.026
LV ESV index	20 (18)	19 (6)	20 (10)	20 (12)	0.921
LVEF	64 (12)	69 (10)	69 (9)	61 (12)	0.014
PA RAC	10 (8)	16 (14)	12 (8)	8 (6)	0.008
VMI	0.43 (0.28)	0.28 (0.11)	0.28 (0.17)	0.51 (0.31)	0.001
IVS angle	165 (24)	134 (10)	152 (14)	174 (20)	<0.001
Black blood score	3 (1)	1 (1)	2 (1)	3 (1)	<0.001
Alpha-index	25 (14)	13 (6)	16 (6)	30 (14)	<0.001
CMR-RV	41 (9)	33 (4)	35 (5)	44 (9)	<0.001
CMR-PA/RV	35 (9)	23 (5)	30 (5)	39 (7)	<0.001

PH: pulmonary hypertension, WHO: World Health Organisation, ISWT: Incremental shuttle walk test, FEV1: Forced expiratory volume in 1 s, RVC-mPAP: right heart catheter measured mean pulmonary artery pressure, mRAP: mean right atrial pressure, PAWP: pulmonary arterial wedge pressure, CI: cardiac index, PVRI: pulmonary vascular resistance index, SvO_2_: mixed venous oxygen saturation, FVC: forced vital capacity, TLCO: transfer factor for carbon monoxide, RHC: right heart catheter, MRI: magnetic resonance imaging

### Diagnostic accuracy

**Fig. 3:**
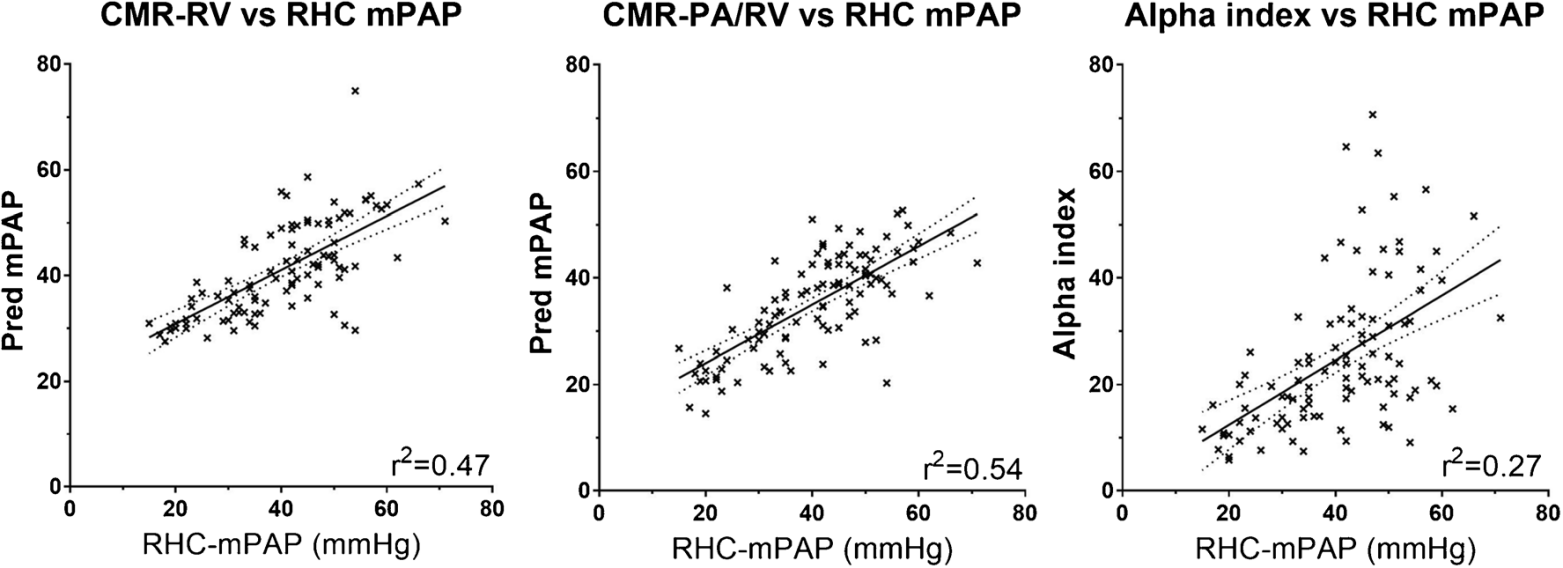
Scatter plots showing correlations of each MRI model with right heart catheter measured mPAP

Table 2 shows the univariate correlations of CMR imaging metrics: RV end diastolic volume index, RV end systolic volume index, RV ejection fraction, RV mass, PA diastolic area and relative area change, ventricular mass index, interventricular septal angle septal angle, average velocity and black blood score all showed statistically significant correlations with RHC mPAP. All of the models showed strong correlations with RHC measured mPAP, with *p*-values <0.0001. The CMR-PA/RV model showed stronger correlation with mPAP than the individual quantitative measurements alone. A scatter plot of the MRI models against RHC-mPAP is given in Fig. 3. The models all showed a stronger correlation with mPAP in the patients with mPAP <35 mmHg (r- and *p*-values for PA-RV were 0.550 and 0.001, CMR-PA/RV 0.653 and <0.001 and alpha 0.341 and 0.052) than in the patients with mPAP ≥35 mmHg (r- and *p*-values for PA-RV were 0.431 and <0.001, CMR-PA/RV 0.407 and 0.001 and alpha 0.210 and 0.083).

**Table 2:** CMR imaging metrics and MRI model correlations with mPAP

	Correlation with mPAP	
	R	*p*-value
RVEDV index	0.377	<0.0001
RVESV index	0.482	<0.0001
RVEF	-0.585	<0.0001
RV mass	0.372	<0.0001
LVEDV index	-0.304	0.002
LVESV index	-0.029	0.772
LVEF	-0.221	0.026
PA diastolic area	0.401	<0.0001
PA RAC	-0.344	<0.0001
VMI	0.470	<0.0001
IVS angle	0.710	<0.0001
Average PA velocity	-0.428	<0.0001
Black blood score	0.603	<0.0001
CMR-RV	0.689	<0.0001
CMR-PA/RV	0.732	<0.0001
Alpha index	0.527	<0.0001

RVEDV: right ventricular end-diastolic volume, RVESV: right ventricular end-systolic volume, RVEF: right ventricular ejection fraction, RV: right ventricle, LVEDV: left ventricular end-diastolic volume, LVESV: left ventricular end-systolic volume, LVEF: left ventricular ejection fraction, PA: pulmonary artery, PA RAC: pulmonary artery relative area change, VMI: ventricular mass index, IVS: interventricular septum

**Fig. 4:**
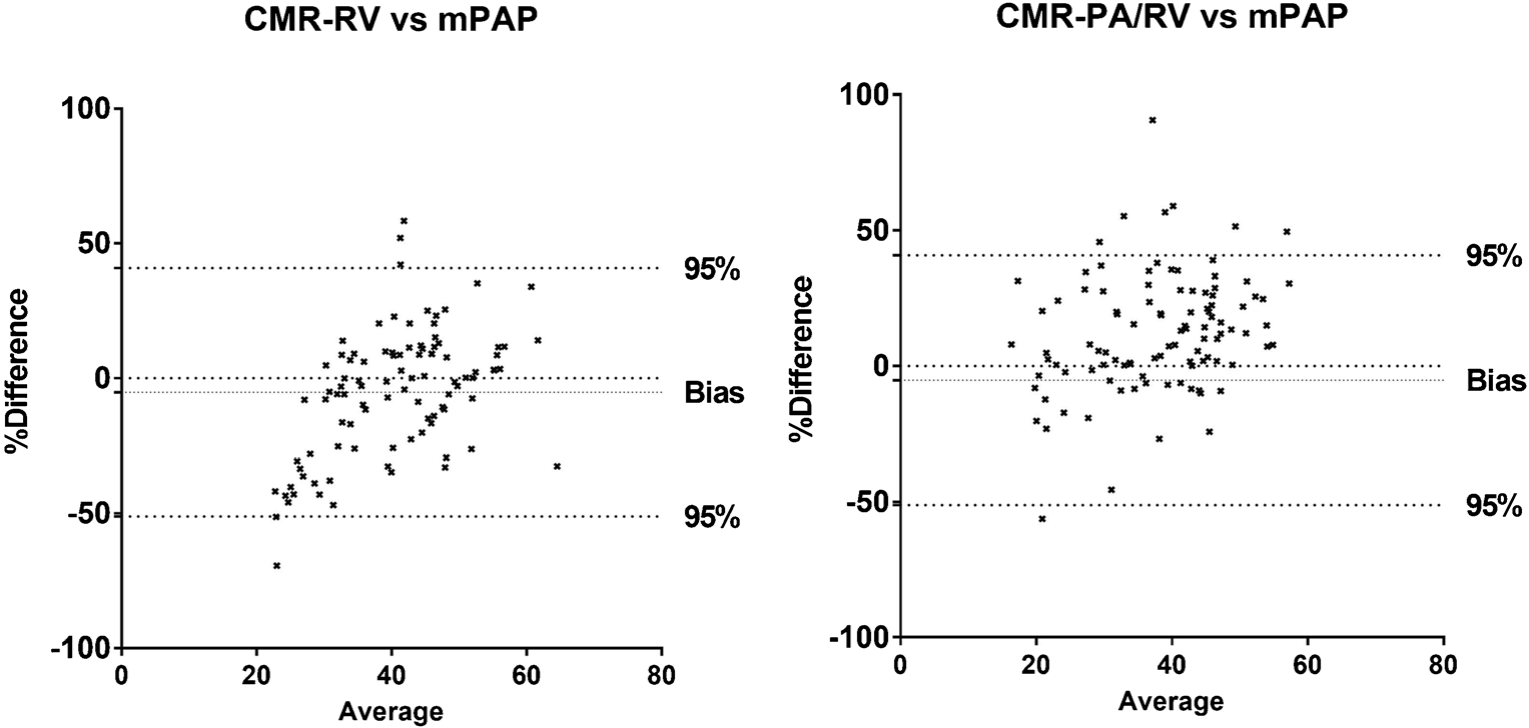
Bland-Altman plots showing accuracy of models against RHC-mPAP

**Fig. 5:**
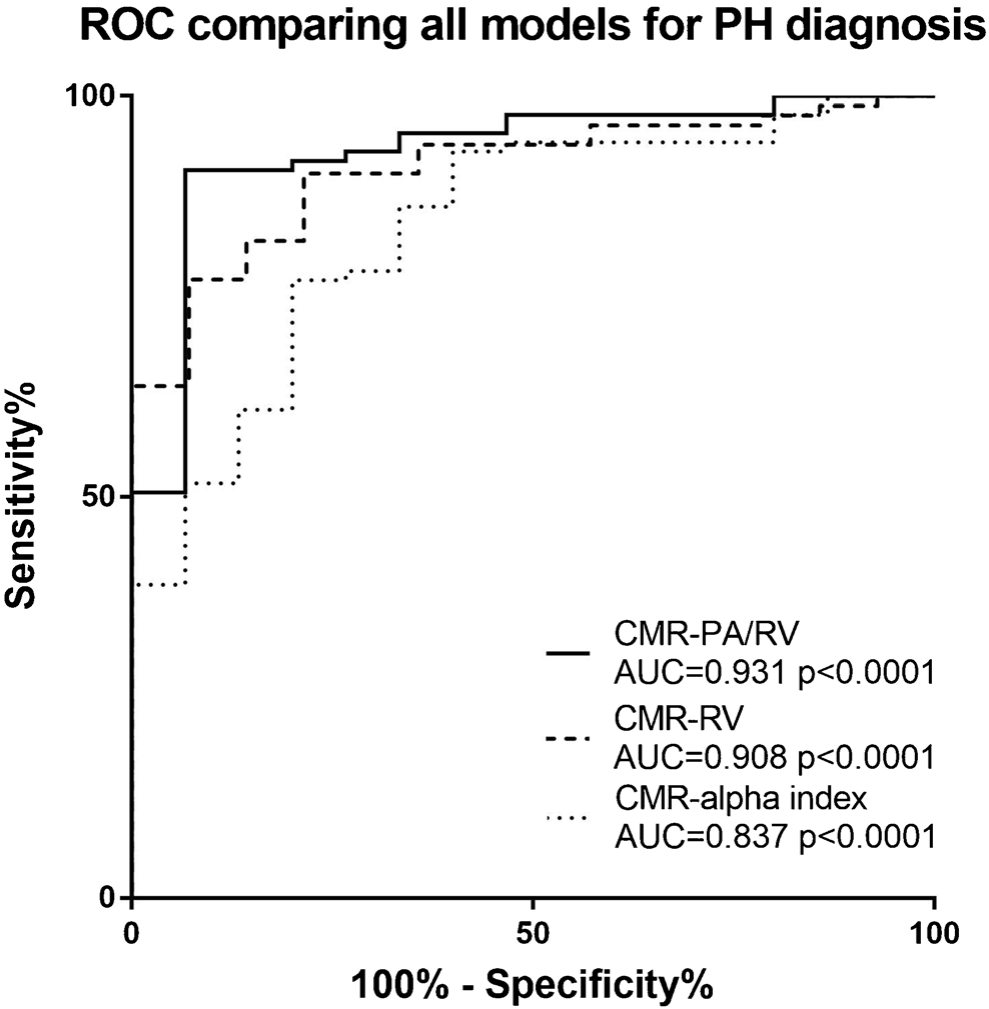
ROC curve for the diagnosis of pulmonary hypertension

Bland-Altman plots were also created to assess the accuracy of the models against RHC-mPAP (Fig. 4), this was not performed for alpha index as it was not designed to directly assess mPAP. Bland-Altman analysis showed modest accuracy for CMR-RV (bias -5.2%, limits of agreement -51.2 to 40.8%) and CMR-PA/RV models (bias 12.2% and limits of agreement -30.7 to 55.0%). Table 2 shows the correlation of the CMR models against RHC mPAP. Figure 5 shows the ROC curves for the three CMR models. CMR-PA/RV and CMR-RV had the largest AUC values, 0.93 (95% confidence interval 0.86-1.0) and 0.91 (95% confidence interval 0.84-0.97), respectively, and alpha index also showed good diagnostic accuracy with AUC 0.837 (95% confidence interval 0.74-0.94). The sensitivity and specificity of the CMR-PA/RV model was 92% and 80%, CMR-RV model 90% and 79%, and the alpha index 100% and 13%, respectively (Table 3).

**Table 3:** Diagnostic accuracy for pulmonary hypertension

	Sensitivity	Specificity	Positive predictive value	Negative predictive value	ROC Area under curve	*p*-value
CMR-RV (threshold 32)	90% (95% CI 82-95)	79% (95% CI 52-92)	96% (95% CI 89-99)	58% (95% CI 36-77%)	0.908 (95% CI 0.84-0.97)	<0.0001
CMR-PA/RV (threshold 25)	92% (95% CI 84-96%)	80% (95% CI 55-93%)	96% (95% CI 90-93%)	63% (95% CI 41-81%)	0.931 (95% CI 0.86-1.00)	<0.0001
Alpha Index (Threshold 7.2)	100% (95%CI 96-100)	13% (95% CI 0-38)	87% (95% CI 79-92%	100% (95% CI 18-100%)	0.837 (95% CI 0.73-0.94)	0.020

### Outcome

**Fig. 6:**
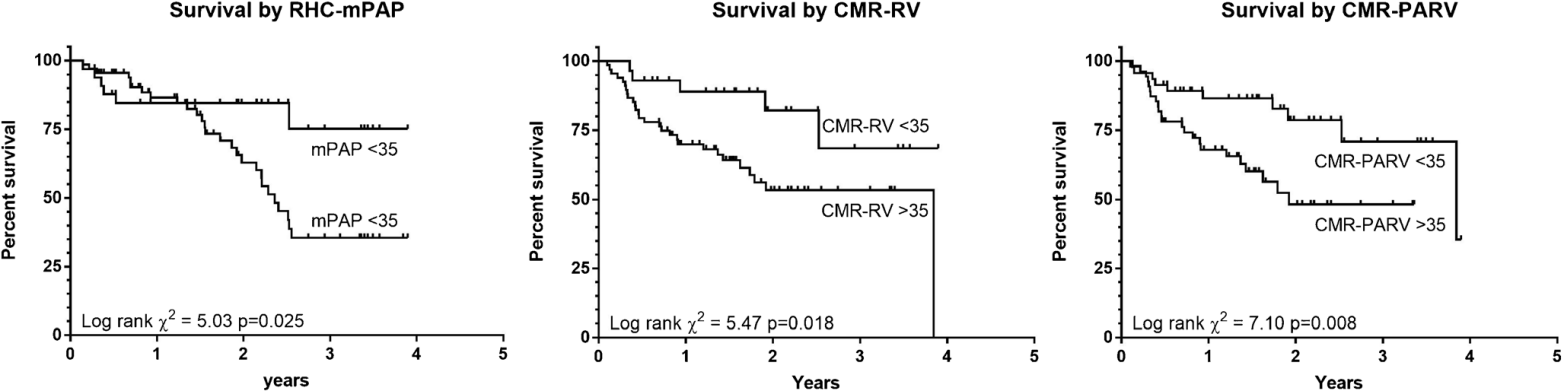
Kaplan-Meier survival tables, all dichotomised by 35

During a mean follow up period of 1.5 years (standard deviation 0.9) there were 33 deaths. Table 4 gives the scaled univariate Cox proportional hazards regression results for patient demographics, RHC and CMR measurements. Age, sex, walk distance FEV1 percent predicted, FVC percent predicted and FEV1/FVC ratio were not univariate predictors of mortality. PA relative area change, right ventricular mass index, ventricular mass index, interventricular septal angle and RV end-diastolic volume index were univariate predictors of mortality (*p*=0.009, *p*<0.001, *p*=0.002 and *p*=0.023, respectively). RHC measured mPAP was a strong predictor of mortality (*p*=0.004), as were the CMR-RV and CMR-PA/RV models (*p*=0.002 and *p*=0.012, respectively). There was a statistically significant difference in survival when the population was split by a threshold of 35 mmHg for RHC-mPAP, CMR-RV and CMR-PA/RV models, with a log rank chi squared of 5.03, 5.47 and 7.10, respectively (see Fig. 6 and Table 5). On multivariate analysis (performed on all univariate predictors of outcome for demographics, right heart catheter and cardiac MRI), right ventricular mass index and PA relative area change were statistically significant, with scaled Cox multivariate hazard ratios 1.549 (95% CI 1.172-2.047, *p*=0.002) and 0.561 (95% CI 0.333-0.946, *p*=0.030), respectively.

**Table 4:** Univariate Cox proportional hazards regression analysis for survival

	Cox univariate hazard ratio	95% confidence interval		*p*-value
Demographics				
Age	1.424	0.936	2.166	0.098
Sex	0.781	0.388	1.57	0.487
WHO functional status	1.45	1.031	2.039	0.033
Walk distance	0.475	0.167	1.354	0.164
FVC % pred	1.221	0.835	1.785	0.304
FEV1 % pred	1.399	0.928	2.108	0.109
FEV1/FVC	1.244	0.85	1.82	0.262
TLCO % pred	0.593	0.19	1.852	0.369
Right heart catheter				
mPAP	1.74	1.198	2.526	0.004
mRAP	1.297	0.873	1.927	0.199
PAWP	0.933	0.651	1.338	0.707
CI	0.847	0.566	1.268	0.421
PVRI	1.384	1.034	1.853	0.029
SvO2	0.857	0.59	1.245	0.418
Cardiac MRI				
RV EDV index	1.381	1.013	1.882	0.041
RV ESV index	1.338	0.981	1.824	0.066
RVEF	0.784	0.548	1.122	0.183
RV mass index	1.673	1.303	2.148	<0.001
LV EDV index	0.759	0.494	1.166	0.209
LV ESV index	0.766	0.478	1.228	0.269
LVEF	1.077	0.756	1.535	0.681
PA RAC	0.543	0.343	0.859	0.009
VMI	1.417	1.135	1.768	0.002
IVS angle	1.48	1.055	2.075	0.023
Black blood score	1.377	0.925	2.05	0.115
Average PA velocity	1.018	0.697	1.484	0.928
Alpha-index	1.19	0.858	1.65	0.297
CMR-RV	1.565	1.175	2.083	0.002
CMR-PA/RV	1.59	1.108	2.282	0.012

WHO: World Health Organisation, FEV1: Forced expiratory volume in 1 s, FVC: Forced vital capacity, RHC: right heart catheter, mPAP: right heart catheter measured mean pulmonary artery pressure, mRAP: mean right atrial pressure, PAWP: pulmonary artery wedge pressure, CI: cardiac index, PVRI: pulmonary vascular resistance index, SvO_2_: mixed venous oxygen saturation, CMR: cardiopulmonary magnetic resonance, RVEDV: right ventricular end-diastolic volume, RVESV: right ventricular end-systolic volume, RVEF: right ventricular ejection fraction, RV: right ventricle, LVEDV: left ventricular end-diastolic volume, LVESV: left ventricular end-systolic volume, LVEF: left ventricular ejection fraction, PA RAC: pulmonary artery relative area change, VMI: ventricular mass index, IVS: interventricular septum, CMR-RV: right ventricle based cardiopulmonary magnetic resonance image model, CMR-PA/RV: pulmonary artery and right ventricle based cardiopulmonary magnetic resonance image model

## Discussion

This study demonstrates that CMR models can be used to assess for the presence of pulmonary hypertension in COPD with good diagnostic accuracy, whilst also providing prognostic information similar to right heart catheterisation. This data supports the potential use of CMR in the non-invasive assessment of suspected pulmonary hypertension in patients with COPD. We have shown that single measurements from cardiac MRI (most notably septal angle) correlate strongly with right heart catheter measured mPAP. A model which includes measurements from the right ventricle, the diagnostic accuracy is slightly reduced, but inclusion of basic measurements of pulmonary arterial structure and function increases the accuracy of predicted mPAP and, therefore, the presence of pulmonary hypertension. Therefore, we recommend the use of CMR-PA/RV model in the diagnosis of pulmonary hypertension in patients with COPD. In the presence of COPD, right ventricular mass index and PA relative area change are the strongest predictors of outcome, but further work to develop prognostic thresholds is required.

Studies performed primarily in patients with pulmonary arterial hypertension have shown that structural and functional indices of the right ventricle (RV end diastolic volume index, RV end-systolic volume index, RV ejection fraction, RV mass, interventricular septal angle) and pulmonary artery (PA area, PA relative area change and black blood score) correlate with RHC measured mPAP. Inter- and intra-observer reproducibility data for these cardiac MRI metrics for DC and AJS have been previously published. RV end-diastolic and systolic measurements had excellent inter-observer reproducibility (ICC 0.959 and 0.991) and pulmonary artery relative area change had high inter-observer reproducibility (ICC 0.891). Right ventricular ejection fraction also had high inter-observer reproducibility (ICC 0.957) [22]. We and others, have shown that we can further develop predictive equations using MR metrics to estimate measurements made at cardiac catheterisation. However, to date there is very little data on the use of MR imaging as a diagnostic and prognostic tool in patients with COPD and suspected pulmonary hypertension. In this study we have shown that these structural and functional indices of the right ventricle and pulmonary artery correlate with right heart catheter measured mean pulmonary arterial pressure: with pulmonary artery relative area change, ventricular mass index and interventricular septal angle having the strongest correlations. We have shown that the use of CMR-PA/RV and CMR-RV models, improve the prediction of mPAP and also the diagnostic accuracy of quantitative CMR measures.

The alpha model showed good correlation with mPAP, but had a low diagnostic accuracy: this model suffered from a diagnostic threshold that was too low for this cohort, significantly reducing its specificity. Changing the threshold of the alpha index to 16 increased the diagnostic accuracy of this model to 77% sensitivity and 80% specificity. This inaccuracy may be related to the measurement of right ventricular ejection fraction, particularly the reproducibility between different centres. In the original paper, it was not described how the right ventricular endocardial contours were measured, specifically, whether the papillary muscles and RV trabeculations were included, which may potentially add a bias into the calculation.

Pulmonary hypertension, confirmed by right heart catheter, has been recognised for many years as a marker of disease severity in COPD. In this study we have shown that cardiac MRI measured right ventricular mass and therefore ventricular mass index, along with pulmonary artery relative area change are significant predictors of mortality on univariate analysis. However, by using CMR derived parametric models, particularly the CMR-RV model which combine these CMRI derived measures, we can improve the prediction of outcome in COPD patients on multivariate analysis. These models may be further improved with novel imaging biomarkers in pulmonary hypertension assessment. One such marker is the 4D flow assessment of the life of vortices within the pulmonary artery, which has been shown to have a very high correlation with mPAP (r^2^ 0.95) [23].

A potential source of error arises from the use of MR models, which were developed for use in a cohort of all causes of pulmonary hypertension and not validated in specific sub-groups, such as PH-COPD in this case. We feel that it is likely that the findings of a raised interventricular septal angle and ventricular mass index are likely to occur most subgroups of pulmonary hypertension (with the exception of left heart disease) as they are related to the pressure differential between the left and right ventricles. The measures of pulmonary arterial structure and function (size and relative area change) are likely to be transferable across subgroups, as they are markers of pulmonary vascular compliance and remodelling. We, therefore, feel that it reasonable to use these models in specific sub-groups of pulmonary hypertension, although validation, such as in this paper, would be useful. The models that are used in this paper use parameters that are stated with a degree of precision (for example an offset of 21.806 for CMR-PA/RV), likely more than is required for this purpose. We have maintained the equations in the published form to reduce any bias in the calculations, but it is likely that fewer decimal places could be used for these parameters for the prediction of outcome and the presence of pulmonary hypertension in COPD.

Currently, RHC is the gold standard test for the assessment of pulmonary hypertension and is used to assess prognosis in suspected COPD-PH cases. The ability to use non-invasive CMR models in this patient group may avoid the need for RHC, as we have shown CMR to be as good in the assessment of prognosis. The strong correlation of CMR imaging models with RHC mPAP suggest it may also have a role in follow-up, and also in assessment of treatment response in possible future trials assessing vasodilator response in COPD-PH. The role of cardiac MRI in COPD is further strengthened as this is a cohort of patients in which echocardiography is challenging. In the presence of COPD echocardiography has a relatively high rate of non-diagnostic quality studies. It has a good negative predictive value, but a poorer positive predictive value [14]. In patients with a normal right ventricle and predicted systolic pulmonary artery pressure (estimated from the tricuspid regurgitant jet velocity using the Bernoulli equation) pulmonary hypertension is highly unlikely. The main role for cardiac MRI in patients with COPD, therefore, probably lies in the group of patients with high estimated systolic pulmonary artery pressures or non-diagnostic scans. This may be at diagnosis, as studied in this paper, or potentially at follow-up.

The non-invasive estimation of mPAP has multiple other potential uses, beyond prognosis in COPD, although these have not been addressed in this paper. In advanced COPD patients considered for lung volume reduction surgery (LVRS), severe pulmonary hypertension is considered a contraindication [24], so the non-invasive assessment of mPAP on MR would be a useful tool in patient selection. Furthermore, left or right ventricular dysfunction is considered a relative contraindication to LVRS [24], and can be assessed at the same CMR sitting. In COPD patients with pulmonary hypertension there is evidence that outcome may be improved if treated with lung transplantation over LVRS [25], assessment of mPAP could be performed in the same sitting as a ventilation study in the preoperative assessment of LVRS or transplant candidates [26, 27], allowing for the assessment of the best surgical option and the best target in the lung.

This study is limited by its single centre, retrospective design, at a tertiary pulmonary hypertension referral centre where the severity of pulmonary hypertension is more severe. The results, therefore, represent a biased population, as the patients have been screened by echocardiography and excessive symptoms for the presence of pulmonary hypertension before referral (the vast majority of patients with COPD are managed in primary care). This has resulted in a smaller proportion of non-pulmonary hypertension patients than would be present in a general population of COPD; however, in the absence of a prospective trial, this is difficult to overcome. Furthermore, the assessment of the presence of COPD in the population relied upon the accurate documentation of a clinical diagnosis of COPD. This likely resulted in a lower proportion of patients being identified, but is likely to be more robust than using either spirometry or CT defined emphysema alone. Despite this, we feel that these tools are valid for the assessment of patients who are referred to a pulmonary hypertension centre regarding the presence of pulmonary hypertension and prognosis, as this is the population that was studied. It may be that more specific diagnostic thresholds would be suitable for use in the general COPD population.

## Conclusion

The use of the CMR-PA/RV model, derived from cardiovascular magnetic resonance imaging in the assessment of COPD allows for accurate, non-invasive estimation of pulmonary artery pressure and the presence of pulmonary hypertension. Right ventricular mass index and pulmonary artery relative area change are most predictive of outcome.

## References

[1] Chaouat A, Naeije R, Weitzenblum E (2008). Pulmonary hypertension in COPD. Eur Respir J.

[2] Wright JL (2005). Pulmonary hypertension in chronic obstructive pulmonary disease: current theories of pathogenesis and their implications for treatment. Thorax.

[3] Andersen KH, Iversen M, Kjaergaard J (2012). Prevalence, predictors, and survival in pulmonary hypertension related to end-stage chronic obstructive pulmonary disease. J Hear Lung Transplant.

[4] Burrows B, Kettel LJ, Niden AH (1972). Patterns of Cardiovascular Dysfunction in Chronic Obstructive Lung Disease. N Engl J Med.

[5] Cooper R, Ghali J, Simmons BE, Castaner A (1991). Elevated Pulmonary Artery Pressure. Chest.

[6] Oswald-Mammosser M, Weitzenblum E, Quoix E (1995). Prognostic Factors in COPD Patients Receiving Long-term Oxygen Therapy: Importance of Pulmonary Artery Pressure. Chest.

[7] Minai OA, Chaouat A, Adnot S (2010). Pulmonary Hypertension in COPD: Epidemiology, Significance, and Management. Chest.

[8] Hyduk A, Croft JB, Ayala C (2005). Pulmonary hypertension surveillance--United States, 1980-2002. MMWR Surveill Summ.

[9] Seeger W, Adir Y, Barberà JA (2013). Pulmonary Hypertension in Chronic Lung Diseases. J Am Coll Cardiol.

[10] Boerrigter BG, Bogaard HJ, Trip P (2012). Ventilatory and Cardiocirculatory Exercise Profiles in COPD. Chest.

[11] Hurdman J, Condliffe R, Elliot CA (2013). Pulmonary hypertension in COPD: results from the ASPIRE registry. Eur Respir J.

[12] Hoeper MM, Lee SH, Voswinckel R (2006). Complications of Right Heart Catheterization Procedures in Patients With Pulmonary Hypertension in Experienced Centers. J Am Coll Cardiol.

[13] Fisher MR, Criner GJ, Fishman AP (2007). Estimating pulmonary artery pressures by echocardiography in patients with emphysema. Eur Respir J.

[14] Arcasoy SM, Christie JD, Ferrari VA (2003). Echocardiographic Assessment of Pulmonary Hypertension in Patients with Advanced Lung Disease. Am J Respir Crit Care Med.

[15] Swift AJ, Rajaram S, Hurdman J (2013). Noninvasive Estimation of PA Pressure, Flow, and Resistance With CMR Imaging. JACC Cardiovasc Imaging.

[16] Swift A, Lungu A, Walker H, Capener D, Hammerton C, Elliot C, Condliffe R, Kiely D, Wild J (2015) Improved diagnostic accuracy of MRI in patients with suspected pulmonary hypertension with combined right ventricle and pulmonary artery metrics. ERS Int Congr 2015

[17] Moral S, Fernández-Friera L, Stevens G (2012). New index alpha improves detection of pulmonary hypertension in comparison with other cardiac magnetic resonance indices. Int J Cardiol.

[18] Hurdman J, Condliffe R, Elliot CA (2012). ASPIRE registry: Assessing the Spectrum of Pulmonary hypertension Identified at a REferral centre. Eur Respir J.

[19] Hansell DM, Bankier AA, MacMahon H (2008). Fleischner Society: Glossary of Terms for Thoracic Imaging. Radiology.

[20] Saba TS, Foster J, Cockburn M (2002). Ventricular mass index using magnetic resonance imaging accurately estimates pulmonary artery pressure. Eur Respir J.

[21] Swift AJ, Rajaram S, Condliffe R (2012). Pulmonary artery relative area change detects mild elevations in pulmonary vascular resistance and predicts adverse outcome in pulmonary hypertension. Invest Radiol.

[22] Swift AJ, Capener D, Johns CS et al (2017) Magnetic Resonance Imaging in the Prognostic Evaluation of Patients with Pulmonary Arterial Hypertension. Am J Respir Crit Care Med. 10.1164/rccm.201611-2365OC 10.1164/rccm.201611-2365OC PMC5519970 28328237

[23] Reiter G, Reiter U, Kovacs G (2015). Blood flow vortices along the main pulmonary artery measured with MR imaging for diagnosis of pulmonary hypertension. Radiology.

[24] DeCamp MM, Lipson D, Krasna M (2008). The Evaluation and Preparation of the Patient for Lung Volume Reduction Surgery. Proc Am Thorac Soc.

[25] Meyers BF (2003). Chronic obstructive pulmonary disease * 10: Bullectomy, lung volume reduction surgery, and transplantation for patients with chronic obstructive pulmonary disease. Thorax.

[26] Bauman G, Puderbach M, Deimling M (2009). Non-contrast-enhanced perfusion and ventilation assessment of the human lung by means of Fourier decomposition in proton MRI. Magn Reson Med.

[27] Swift AJ, Wild JM, Fichele S (2005). Emphysematous changes and normal variation in smokers and COPD patients using diffusion 3He MRI. Eur J Radiol.

